# Cost‐of‐illness study for axillary hyperhidrosis in Japan

**DOI:** 10.1111/1346-8138.16050

**Published:** 2021-07-09

**Authors:** Hiroyuki Murota, Tomoko Fujimoto, Yuichiro Oshima, Yasuhiko Tamada, Takeshi Yanagishita, Naoya Murayama, Sachie Inoue, Hiromichi Okatsu, Hiroshi Miyama, Hiroo Yokozeki

**Affiliations:** ^1^ Department of Dermatology Nagasaki University Nagasaki Japan; ^2^ Ikebukuro Nishiguchi Fukurou Dermatology Clinic Tokyo Japan; ^3^ Department of Dermatology Aichi Medical University School of Medicine Aichi Japan; ^4^ CRECON Medical Assessment Inc. Tokyo Japan; ^5^ Medical Affairs Department Kaken Pharmaceutical Co., Ltd. Tokyo Japan; ^6^ Department of Dermatology Tokyo Medical and Dental University Tokyo Japan

**Keywords:** axillary hyperhidrosis, cost of illness, direct medical cost, overall work impairment, self‐medication cost

## Abstract

The prevalence of primary axillary hyperhidrosis in Japan is 5.75% (males, 6.60%; females, 4.72%) in the population aged 5–64 years. No study on comprehensively evaluated direct medical costs, hygiene product costs, and productivity loss in axillary hyperhidrosis patients has been published in Japan. The aim of this study was to estimate the cost of illness for axillary hyperhidrosis in Japan by conducting a nationwide insurance claims database analysis and a cross‐sectional Web‐based survey. Among patients diagnosed with primary axillary hyperhidrosis at least once between November 2012 and October 2019, health insurance receipt data of 1447 patients were analyzed. A cross‐sectional Web‐based survey was conducted on 321 patients aged 16–59 years with axillary hyperhidrosis to calculate hygiene product costs and productivity loss using a Work Productivity and Activity Impairment questionnaire. Furthermore, nationwide estimation was performed for the hygiene product costs and productivity loss based on the number of patients estimated from the prevalence. The annual direct medical costs per axillary hyperhidrosis patient were ¥91 491 in 2016, ¥93 155 in 2017, and ¥75 036 in 2018. In all of these years, botulinum toxin type A injection accounted for approximately 90% of the total costs. The annual total cost of hygiene products per axillary hyperhidrosis patient was ¥9325. The overall work impairment (%) of working patients with axillary hyperhidrosis was 30.52%, and its monthly productivity loss was ¥120 593/patient. The activity impairment (%) of full‐time housewives with axillary hyperhidrosis was 49.05% and its monthly productivity loss was ¥176 368/patient. The annual hygiene product cost based on the nationwide estimation was ¥24.5 billion and the monthly productivity loss was ¥312 billion. The significant cost associated with axillary hyperhidrosis was clarified. If out‐of‐pocket expenses for treatments not covered by health insurance are included in the estimation, the cost will further increase.

## 1 INTRODUCTION

Primary axillary hyperhidrosis, a disease of unknown pathogenesis with no underlying disease, interferes with daily life because of excessive axillary sweating. It is primary focal hyperhidrosis classified as an intractable disease. A questionnaire survey conducted by Fujimoto *et al*.^1^ in a Japanese population aged 5–64 years (5807 valid responses) revealed the prevalence of primary axillary hyperhidrosis to be 5.75% (6.60% and 4.72% in males and females, respectively) between December 2009 and January 2010. The distribution of Hyperhidrosis Disease Severity Scale (HDSS) scores of 1, 2, and 3 or 4 (severe) was 5.6%, 47.6%, and 46.8%, respectively, suggesting that nearly half of the respondents had severe symptoms.

Cost‐of‐illness studies aim to identify and measure all of the costs of a particular disease, including the direct, indirect, and intangible dimensions. Direct costs consist of health‐care costs and non‐health‐care costs like for transportation and household expenditure. Indirect cost generally refers to lost productivity resulting from morbidity or mortality in pharmacoeconomics and outcomes research area. Intangible cost is the costs assigned to the amount of suffering that occurs because of the disease or health‐care intervention.^2^ It is widely believed that estimating the total societal cost of an illness is a useful aid to policy making, and organizations such as the World Bank and the World Health Organization commonly use such studies.^3^, ^4^, ^5^, ^6^ Many cost‐of‐illness studies targeted for various countries have been reported in the field of dermatology.^7^, ^8^, ^9^, ^10^ Axillary hyperhidrosis causes psychological burdens, such as depression, loss of self‐confidence, and weakness,^11^ suggesting significant emotional burdens. Moreover, a Work Productivity and Activity Impairment (WPAI) survey of hyperhidrosis patients who visited a university hospital demonstrated the presenteeism of hyperhidrosis patients to be 47.14%, suggesting the significant effects of hyperhidrosis on work performance. The productivity loss associated with hyperhidrosis in Japan in 2009 was estimated to be ¥197 billion/month.^12^

As axillary hyperhidrosis is treated at one’s own expense and by health insurance, only limited information is available on treatment methods and medical costs. Furthermore, little information is available on the costs of so‐called self‐medication, namely hygiene products such as sweat pads and antiperspirants.

The aim of this study was to estimate the cost associated with axillary hyperhidrosis in Japan by conducting a nationwide insurance claims database analysis and a cross‐sectional Web‐based survey.

## 2 METHODS

### 2.1 Study design

Direct medical costs, hygiene product costs, and productivity loss were estimated as a cost associated with axillary hyperhidrosis in Japan. Direct medical costs were estimated using a nationwide insurance claims database provided by JMDC.^13^ Hygiene product costs and productivity loss were estimated using the results of the cross‐sectional Web‐based survey.

### 2.2 Direct medical costs

Direct medical costs were estimated using the receipt data from 31 412 patients who made at least one visit for hyperhidrosis (the corresponding ICD‐10, International Classification of Diseases, 10th Revision, and the name of illness are shown in Table 1) between January 2005 and October 2019. Among the 1536 patients diagnosed with primary axillary hyperhidrosis at least once between November 2012 and October 2019 after the expansion of the indications for botulinum toxin type A injection (Botox^®^; GlaxoSmithKline), those diagnosed with hyperhidrosis at different sites (primary systemic hyperhidrosis, systemic hyperhidrosis, primary palmoplantar hyperhidrosis, palmoplantar hyperhidrosis, palmar hyperhidrosis, plantar hyperhidrosis, facial hyperhidrosis, and nervous hyperhidrosis) between January 2005 and October 2019 were excluded, and the remaining 1447 for whom health insurance receipt data were available were included in the analysis.

**TABLE 1:** ICD‐10 codes and names of illnesses related to hyperhidrosis

ICD‐10 codes	Names of illnesses
F45	Nervous hyperhidrosis^a^
R61	Facial hyperhidrosis^a^
R61	Focal hyperhidrosis^b^
R61	Primary focal hyperhidrosis^b^
R61	Primary palmoplantar hyperhidrosis^a^
R61	Primary axillary hyperhidrosis^b,c^
R61	Palmar hyperhidrosis^a^
R61	Palmoplantar hyperhidrosis^a^
R61	Plantar hyperhidrosis^a^
R61	Axillary hyperhidrosis^b^
R61	Primary systemic hyperhidrosis^a^
R61	Systemic hyperhidrosis^a^
R61	Hyperhidrosis^b^

Abbreviation: ICD‐10, International Classification of Diseases, 10th Revision.

Selection criteria for the analysis population were: patients diagnosed with primary axillary hyperhidrosis^c^ at least once between November 2012 and October 2019 after the expansion of indications for botulinum toxin type A injection.

Exclusion criteria were: patients diagnosed with hyperhidrosis^a^ at other sites (primary systemic hyperhidrosis, systemic hyperhidrosis, primary palmoplantar hyperhidrosis, palmoplantar hyperhidrosis, palmar hyperhidrosis, plantar hyperhidrosis, facial hyperhidrosis, and nervous hyperhidrosis) between January 2005 and October 2019 for whom data were available.

Receipt data for analysis: receipts for the diagnoses of axillary hyperhidrosis or non‐site‐specific hyperhidrosis (axillary hyperhidrosis, focal hyperhidrosis, primary axillary hyperhidrosis, primary focal hyperhidrosis, and hyperhidrosis).^b^

Medical costs associated with axillary hyperhidrosis were defined as the total costs of receipts for the diagnoses of axillary hyperhidrosis or non‐site‐specific hyperhidrosis (primary axillary hyperhidrosis, axillary hyperhidrosis, primary focal hyperhidrosis, focal hyperhidrosis, and hyperhidrosis) among all receipts in the 1447 analysis population. The annual total medical costs associated with axillary hyperhidrosis were calculated by fiscal year (FY) from 2016 to 2018. The annual total medical costs, including drug costs (e.g. botulinum toxin type A injection), examination costs (Code D of the Medical Fee Classification), surgery costs (Code K of the Medical Fee Classification), and medical treatment costs (Codes other than D and K of the Medical Fee Classification), were separately calculated.

### 2.3 Self‐medication costs and productivity loss

Hygiene product costs and productivity loss in 321 patients aged 16–59 years with HDSS score 2 or above (adjusting HDSS 2:3:4 to approximately 1:1:1) who met the diagnostic criteria for primary axillary hyperhidrosis were estimated based on the cross‐sectional Web‐based survey conducted in April 2020.

Hygiene product costs were defined as annual costs for purchasing clothes/towels, sweat pads, antiperspirants (sprays, sheets, and creams), body cleansers (soap and body soap), foods, and supplements.

Using a WPAI survey questionnaire which is a well‐validated instrument to measure impairments in work and activities,^14^ productivity loss was determined as absenteeism (%), presenteeism (%), and overall work impairment (OWI) (%) in working patients with axillary hyperhidrosis and activity impairment (AI) (%) in full‐time housewives with axillary hyperhidrosis. To calculate productivity loss in working patients with axillary hyperhidrosis, each score was multiplied by monthly wages by sex and age,^15^ and converted into productivity loss (¥/month). To calculate productivity loss in full‐time housewives with axillary hyperhidrosis, each score was multiplied by the monthly housework activity evaluation value^16^ and converted into productivity loss (¥/month).

In general, to estimate productivity loss, annual costs are calculated on the assumption that such productivity loss based on a WPAI survey continues for 1 year. However, seasonal fluctuations in productivity loss should be considered in view of the characteristics of hyperhidrosis. Therefore, to avoid overestimation/underestimation due to the fluctuations, the estimation period was set as 1 month.

### 2.4 Nationwide estimation

Nationwide estimations were performed on hygiene product costs and productivity loss in axillary hyperhidrosis patients with HDSS 2 or above.

#### 2.4.1 Estimation of the number of patients

To calculate the numbers of axillary hyperhidrosis patients by sex and HDSS, populations (31 325 000 males, 30 406 000 females)^17^ aged 20–59 years were multiplied by the prevalence of axillary hyperhidrosis, namely 6.60% and 4.72% in males and females, respectively,^1^ and additionally by the HDSS rates of primary focal hyperhidrosis (HDSS 1, 5.68%; HDSS 2, 46.11%; HDSS 3, 35.58%; and HDSS 4, 12.63% in males; and HDSS 1, 5.31%; HDSS 2, 50.61%; HDSS 3, 32.65%; and HDSS 4, 11.43% in females).^18^

To calculate the numbers of working patients by sex and HDSS, the numbers of workers aged 20–59 (28 190 000 males and 23 300 000 females)^19^ were multiplied by the prevalence of axillary hyperhidrosis and by the HDSS rates of primary focal hyperhidrosis. To calculate the numbers of full‐time housewives with axillary hyperhidrosis and HDSS, the number of full‐time housewives aged 20–59 (5 907 258 persons)^17^, ^20^ was multiplied similarly.

#### 2.4.2 Self‐medication costs

Hygiene products costs (¥/year) per patient by sex and HDSS adjusted for age distribution were estimated by multiplying the annual hygiene product costs (¥) per patient by sex, age, and HDSS obtained in the Web‐based survey by the patient age distribution (20s, 25.1%; 30s, 30.9%; 40s, 31.7%; and 50s, 12.3%) (Web‐based survey 2017 by Macromill and inhouse data of Kaken Pharmaceutical). The obtained hygiene product costs were multiplied by the total numbers of patients by sex and HDSS to estimate nationwide hygiene product costs (¥/year).

#### 2.4.3 Productivity loss

##### Productivity loss for OWI

The productivity losses (¥/month) per patient, corresponding to OWI (%), by sex and HDSS, adjusted by statuses of employment and age distributions, were estimated by multiplying the productivity loss (¥/month) per patient by sex, statuses of employment, age, and HDSS obtained in the Web‐based survey by the rates of statuses of employment by age of all workers (Labor Force Survey 2019) and the age distribution of patients (inhouse data from the Web‐based survey conducted by Macromill in 2017). The obtained productivity loss was multiplied by the numbers of working patients by sex and HDSS to estimate the nationwide productivity loss (¥/month), corresponding to OWI.

##### Productivity loss for AI

The productivity loss (¥/month) per patient, corresponding to AI (%), by HDSS adjusted by age distribution was estimated by multiplying the productivity loss (¥/month) per patient by age and HDSS obtained in the Web‐based survey by the age distribution of patients. The resulting productivity loss was multiplied by the number of full‐time housewives by HDSS to estimate the nationwide productivity loss (¥/month), corresponding to AI.

## 3 RESULTS

### 3.1 Direct medical costs

The characteristics of the analysis population are summarized in Table 2. Males accounted for 28.8% of all and mean age was 35.7 years at the earliest diagnosis of hyperhidrosis. The age distribution exhibited peaks at 30s and 40s (27.9% and 28.1%, respectively). Of the earliest diagnoses of hyperhidrosis, primary axillary hyperhidrosis was the most common (91.2%). Of the analysis population, 1074 (74%) received at least one dose of botulinum toxin type A injection.

**TABLE 2:** Patient background in analysis population of health insurance receipt data

Items	Values (n = 1447)
Sex (male %)	417 (28.8%)
Age (mean ± SD)^a^	35.7 ± 11.5
Age distribution^a^	
10–19 years	120 (8.3%)
20–29 years	350 (24.2%)
30–39 years	404 (27.9%)
40–49 years	407 (28.1%)
50–59 years	146 (10.1%)
60–69 years	20 (1.4%)
Oldest diagnosis of hyperhidrosis (multiple answers allowed)	
Focal hyperhidrosis	10 (0.7%)
Primary focal hyperhidrosis	3 (0.2%)
Primary axillary hyperhidrosis	1320 (91.2%)
Hyperhidrosis	142 (9.8%)
Axillary hyperhidrosis	62 (4.3%)

Abbreviation: SD, standard deviation.

^a^ Age at the oldest diagnosis of hyperhidrosis.

The annual direct medical costs per axillary hyperhidrosis patient were ¥91 491 in 2016, ¥93 155 in 2017, and ¥75 036 in 2018. Drug costs for botulinum toxin type A injection accounted for approximately 90% of the total costs. They decreased in 2018 compared with the previous year, probably because of the drug price revision for botulinum toxin type A injection.

### 3.2 Self‐medication costs

The patient backgrounds revealed in the Web‐based survey are summarized in Table 3. Females accounted for 60.1% of all and the age distribution peaked at 40s (43.9%). The most common status of employment was regular worker (33.6%), followed by temporary or part‐time worker (27.1%).

**TABLE 3:** Patient background in the Web‐based survey

	Total		HDSS 2		HDSS 3		HDSS 4	
	n	%	n	%	n	%	n	%
Whole population	321	100.0	107	100.0	108	100.0	106	100.0
Sex								
Male	128	39.9	47	43.9	35	32.4	46	43.4
Female	193	60.1	60	56.1	73	67.6	60	56.6
Age distribution								
≥29 years	29	9.0	14	13.1	7	6.5	8	7.5
30–39 years	81	25.2	20	18.7	32	29.6	29	27.4
40–49 years	141	43.9	52	48.6	49	45.4	40	37.7
50–59 years	70	21.8	21	19.6	20	18.5	29	27.4
Status of employment								
Regular worker	108	33.6	35	32.7	41	38.0	32	30.2
Company executive	5	1.6	2	1.9	1	0.9	2	1.9
Self‐employed worker or family worker	17	5.3	6	5.6	4	3.7	7	6.6
Temporary staff or part‐time worker	87	27.1	31	29.0	28	25.9	28	26.4
Full‐time housewife/househusband	44	13.7	14	13.1	18	16.7	12	11.3
Displaced/unemployed	49	15.3	16	15.0	12	11.1	21	19.8
Student	5	1.6	2	1.9	2	1.9	1	0.9
Others	6	1.9	1	0.9	2	1.9	3	2.8

Abbreviation: HDSS, Hyperhidrosis Disease Severity Scale.

The annual total cost of hygiene products per axillary hyperhidrosis patient was ¥9325 (Table 4). The highest cost (¥13 786) per purchaser was associated with foods and supplements. The annual total cost of hygiene products by sex and HDSS was ¥10 510 in males, being higher than the ¥8539 in females. Both males and females spent more at a higher HDSS level.

**TABLE 4:** Annual hygiene product costs (¥/patient) in patients with axillary hyperhidrosis (by sex and HDSS)

Items		HDSS	n	Annual hygiene product cost (¥/patient)		
				Mean	SD	Median
Overall population		–	321	9325	22 452	4000
Breakdown:	Clothes/towels,^a^ and sweat pads		135	6125	10 709	3000
	Antiperspirants (spray, sheets, and cream)		265	5455	11 808	3000
	Body cleansers (soap and body soap)		137	3613	3484	2000
	Foods and supplements		14	13 786	28 397	4500
	Others		5	6620	13 080	1000
Male		Total	128	10 510	31 475	3750
		2	47	5785	13 625	2500
		3	35	10 957	17 270	5500
		4	46	14 999	48 339	5000
Female		Total	193	8539	13 539	4500
		2	60	4884	5094	3000
		3	73	9226	12 251	4500
		4	60	11 360	19109	6000

Abbreviations: HDSS, Hyperhidrosis Disease Severity Scale; SD, standard deviation.

^a^ Newly purchased or replaced clothes because of sweating.

### 3.3 Productivity losses

The OWI (%) in working patients with axillary hyperhidrosis was 30.52% (Table 5). It was 32.18% in females, being higher than the 29.03% in males. Both males and females had a slightly higher OWI (%) in regular workers (29.66% and 33.18%, respectively). Patients with HDSS 3–4 had a slightly higher OWI (%) than those with HDSS 2.

**TABLE 5:** Productivity loss (%) and monthly productivity loss (¥/patient) in patients with axillary hyperhidrosis (by sex and HDSS)

	Sex	Status of employment^a^	HDSS	n	Productivity loss (%)			Monthly productivity loss (¥/patient)		
					Mean	SD	Median	Mean	SD	Median
OWI	Overall population	–	–	195	30.52	30.39	20.00	120 593	139 027	72 127
	Male	Total	–	103	29.03	31.26	20.00	146 164	166 750	95 583
		Regular	Total	88	29.66	31.96	20.00	159 328	174 656	103 900
			2	30	12.74	21.99	0.00	70 005	127 425	0
			3	27	39.30	30.53	30.00	205 773	163 947	180 008
			4	31	37.64	35.38	30.00	205 316	193 307	183 370
		Non‐regular	Total	15	25.33	27.48	10.00	68 941	74 429	27 924
			2	8	8.75	13.56	5.00	24 890	39 488	13 641
			3	2	40.00	28.28	40.00	110 978	74 547	110 978
			4	5	46.00	30.50	60.00	122 607	83 104	130 740
	Female	Total	–	92	32.18	29.47	25.00	91 965	92 035	65 608
		Regular	Total	38	33.18	29.25	30.00	125 819	113 204	111 631
			2	12	25.90	26.20	25.00	101 851	104 824	100 621
			3	17	25.29	24.01	20.00	94 444	91 293	79 603
			4	9	57.78	31.14	60.00	217 040	123 196	238 810
		Non‐regular	Total	54	31.48	29.87	20.00	68 142	64 785	43 738
			2	18	18.33	19.78	15.00	39 013	41 756	32 725
			3	20	38.50	25.81	30.00	83 739	56 126	65 608
			4	16	37.50	39.41	20.00	81 415	85 635	42 995
Absenteeism	Overall population	–	–	195	0.49	3.30	0.00	2011	11 288	0
	Male	Total	–	103	0.52	2.28	0.00	2883	12 906	0
		Regular	Total	88	0.60	2.45	0.00	3374	13 915	0
			2	30	0.09	0.49	0.00	330	1810	0
			3	27	0.09	0.48	0.00	519	2698	0
			4	31	1.55	3.95	0.00	8805	22 471	0
		Non‐regular	Total	15	0.00	0.00	0.00	0	0	0
			2	8	0.00	0.00	0.00	0	0	0
			3	2	0.00	0.00	0.00	0	0	0
			4	5	0.00	0.00	0.00	0	0	0
	Female	Total	–	92	0.46	4.17	0.00	1036	9118	0
		Regular	Total	38	0.05	0.33	0.00	214	1318	0
			2	12	0.17	0.59	0.00	677	2345	0
			3	17	0.00	0.00	0.00	0	0	0
			4	9	0.00	0.00	0.00	0	0	0
		Non‐regular	Total	54	0.74	5.44	0.00	1614	11 861	0
			2	18	0.00	0.00	0.00	0	0	0
			3	20	0.00	0.00	0.00	0	0	0
			4	16	2.50	10.00	0.00	5448	21 791	0
Presenteeism	Overall population	–	–	195	30.03	29.83	20.00	118 582	137 371	69 238
	Male	Total	–	103	28.51	30.99	20.00	143 282	164 906	87 398
		Regular	Total	88	29.06	31.66	20.00	155 954	172 846	103 900
			2	30	12.65	21.96	0.00	69 675	127 408	0
			3	27	39.20	30.46	30.00	205 254	163 521	180 008
			4	31	36.10	34.98	30.00	196 511	190 902	183 370
		Non‐regular	Total	15	25.33	27.48	10.00	68 941	74 429	27 924
			2	8	8.75	13.56	5.00	24 890	39 488	13 641
			3	2	40.00	28.28	40.00	110 978	74 547	110 978
			4	5	46.00	30.50	60.00	122 607	83 104	130 740
	Female	Total	–	92	31.73	28.72	25.00	90 929	91 024	65 608
		Regular	Total	38	33.13	29.20	30.00	125 605	112 986	111 631
			2	12	25.73	25.95	25.00	101 174	103 858	100 621
			3	17	25.29	24.01	20.00	94 444	91 293	79 603
			4	9	57.78	31.14	60.00	217 040	123 196	238 810
		Non‐regular	Total	54	30.74	28.61	20.00	66 527	62 010	43 738
			2	18	18.33	19.78	15.00	39 013	41 756	32 725
			3	20	38.50	25.81	30.00	83 739	56 126	65 608
			4	16	35.00	36.33	20.00	75 967	78 878	42 995
AI^b^	Female	–	Total	42	49.05	32.79	50.00	176 368	124 117	187 333
			2	13	35.39	29.33	40.00	121 185	100 851	139 433
			3	18	57.22	28.66	60.00	207 095	108 967	207 375
			4	11	51.82	41.43	40.00	191 302	157 691	153 400

Abbreviations: ; AI, activity impairment; HDSS, Hyperhidrosis Disease Severity Scale; OWI, overall work impairment; SD, standard deviation; WPAI, Work Productivity and Activity Impairment.

WPAI Questionnaire:

Q1. During the past 7 days, how many hours did you miss from work because of problems associated with your underarm sweating?

Q2. During the past 7 days, how many hours did you actually work?

Q3. During the past 7 days, how much did your underarm sweating affect your productivity while you were working? (Answered on a 0–10 score, with 0 = underarm sweating had no effect on my work and 10 = underarm sweating completely prevented me from working.)

Q4. During the past 7 days, how much did your underarm sweating affect your ability to perform your normal daily activities, other than work at a job? (Answered on a 0–10 score, with 0 = underarm sweating had no effect on my daily activities and 10 = underarm sweating completely prevented me from doing my daily activities.)

Calculating productivity loss (%):

OWI = Q1 / (Q1 + Q2) + (1 − Q1 / [Q1 + Q2]) * (Q3/10).

Absenteeism = Q1 / (Q1 + Q2).

Presenteeism = (1 − Q1 / [Q1 + Q2]) * (Q3 / 10).

AI = Q4 / 10.

^a^ Statuses of employment are as follows: “regular”, regular worker, company executive, and self‐employed worker and family worker; “non‐regular”, temporary staff and part‐time worker.

^b^ Since there were only two males, AI was not calculated for males.

Absenteeism (%) and presenteeism (%) of working patients with axillary hyperhidrosis were 0.49% and 30.03%, respectively. Presenteeism accounted for the majority of OWI. The AI (%) of full‐time housewives with axillary hyperhidrosis was 49.05%. Patients with HDSS 3–4 had a slightly higher AI (%) than those with HDSS 2.

The monthly productivity loss per patient, corresponding to OWI (%), was ¥120 593 (Table 5). The loss in males was ¥146 164, being higher than the ¥91 965 in females. The monthly productivity loss per patient, corresponding to absenteeism (%) and presenteeism (%), was ¥2011 and ¥118 582, respectively. The monthly productivity loss per patient, corresponding to AI (%), was ¥176 368. As observed for OWI (%) and AI (%), the productivity loss was slightly higher in patients with HDSS 3–4 than in those with HDSS 2.

### 3.4 Nationwide estimation

Nationwide estimates in axillary hyperhidrosis patients with HDSS 2 or above are summarized in Table 6.

**TABLE 6:** Nationwide estimated costs of illness associated with axillary hyperhidrosis

	Per axillary hyperhidrosis patient (¥)		Nationwide estimation (¥ billion)		
	Male	Female	Male	Female	Total
Hygiene product cost (¥/year)					
HDSS 2	5944	4779	5.7	3.5	9.1
HDSS 3	9458	9300	7	4.4	11.3
HDSS 4	8918	10 483	2.3	1.7	4
Total	–	–	15	9.5	24.5
Productivity loss (¥/month)					
OWI^a^					
HDSS 2	43 913	56 918	37.7	31.7	69.4
HDSS 3	166 711	82 768	110.4	29.7	140.1
HDSS 4	172 824	140 131	40.6	17.6	58.2
Total	–	–	188.6	79	267.7
AI^a^					
HDSS 2	–	134 266	–	18.9	18.9
HDSS 3	–	226 370	–	20.6	20.6
HDSS 4	–	150 810	–	4.8	4.8
Total	–	–	–	44.4	44.4
Total	–	–	188.6	123.4	312.0

Abbreviations: AI, activity impairment; HDSS, Hyperhidrosis Disease Severity Scale; OWI, overall work impairment.

^a^ Productivity loss was calculated as below.

Wage (¥/month) = ( ([cash wage [including overtime pay] [monthly]) × 12 + (annual bonus and other special wages [annual])) / 12.

OWI = wage (¥/month) × OWI (%).

AI = household activity evaluation (¥/month) × AI (%).

The annual hygiene product cost was ¥24.5 billion (¥15 and ¥9.5 billion in males and females, respectively). The estimated costs by HDSS were ¥9.1 billion for HDSS 2, ¥11.3 billion for HDSS 3, and ¥4 billion for HDSS 4.

The monthly productivity loss, corresponding to OWI (%), was ¥267.7 billion (¥188.6 and ¥79 billion in males and females, respectively). The estimated costs by HDSS were ¥69.4 billion for HDSS 2, ¥140.1 billion for HDSS 3, and ¥58.2 billion for HDSS 4. The monthly productivity loss, corresponding to AI (%), was ¥44.4 billion. The estimated costs by HDSS were ¥18.9 billion for HDSS 2, ¥20.6 billion for HDSS 3, and ¥4.8 billion for HDSS 4. The monthly productivity loss for axillary hyperhidrosis in Japan was estimated to be ¥312 billion (¥188.6 and ¥123.4 billion in males and females, respectively).

## 4 DISCUSSION

In the present study, the cost associated with axillary hyperhidrosis in Japan was estimated by conducting a nationwide insurance claims database analysis for direct medical costs, and a cross‐sectional Web‐based survey for hygiene product costs and productivity loss.

As a result, the direct medical cost required for axillary hyperhidrosis was estimated to be ¥75 036/year (FY 2018) per patient. The use of botulinum toxin type A injection is limited for only severe cases of primary axillary hyperhidrosis under universal health insurance coverage in Japan. Health insurance‐covered treatment using botulinum toxin type A injection accounts for only 20% (doctors in private practice) and 33% (doctors in public practice) in Japan.^21^ Therefore, many patients visit a department of cosmetic dermatology at their own expense, suggesting that the actual medical costs are even higher.

The hygiene product cost per patient was ¥9325/year (¥10 510 and ¥8539/year in males and females, respectively), suggesting that males spend more than females. The cost slightly increased at a higher HDSS level. As productivity loss, the absenteeism (%) and presenteeism (%) of working patients with axillary hyperhidrosis were 0.49% and 30.03%, respectively, suggesting approximately 30% decrease in work performance due to symptoms. The performance was slightly lower in patients with HDSS 3–4 than in those with HDSS 2. Based on these results, the annual hygiene product cost for axillary hyperhidrosis was estimated to be ¥24.5 billion, and the societal loss associated with the productivity loss (OWI [%] and AI [%]) to be ¥312 billion/month.

Hamm *et al*.^22^ examined disease characteristics and functional impairment using the Hyperhidrosis Impact Questionnaire, Dermatology Life Quality Index, and 12‐item Short Form Health Survey in 345 hyperhidrosis patients, revealing that primary hyperhidrosis affected productivity, daily activity, patient well‐being, and human relationship formation. The WPAI survey by Yokozeki *et al*.^12^ demonstrated the same tendency (0% absenteeism and 47.14% presenteeism) as our survey, although their findings cannot be simply compared to ours because their survey was conducted 10 years ago on a wider range of patients with hyperhidrosis, including axillary hyperhidrosis, who visited a university hospital.

In Japan, several reports have been published on the estimated societal loss associated with productivity loss based on a WPAI survey. Working patients with hyperhidrosis had a 46.8% reduction in productivity, with more severe disabilities due to urticaria (33.8%), atopic dermatitis (38.7%), eczema/dermatitis (41.0%), and psoriasis (26.7%).^23^ Murota *et al*.^24^ recently conducted a WPAI survey on 400 patients with atopic dermatitis in Japan, and noted absenteeism of 2.1–5.5% and presenteeism of 31.4–31.8%, demonstrating similarly declined work performance to that observed in our survey. Furthermore, the annual societal loss associated with productivity loss was ¥2.5 trillion (approximately ¥208.3 billion/month). Igarashi *et al*.^25^ reported presenteeism of 32.73% and absenteeism of 6.95% in patients diagnosed with migraine, and the same decline in work performance in axillary hyperhidrosis patients as observed in our survey. Sruamsiri *et al*.^26^ conducted a Web‐based survey on 500 patients with rheumatoid arthritis in Japan, and reported presenteeism of 23% and absenteeism of 1%. As previously reported, we quantitatively demonstrated a significant societal loss associated with axillary hyperhidrosis.

Regarding direct medical costs, medical costs for treatments covered by health insurance can be comprehended using a nationwide insurance claims database. However, the percentage of axillary hyperhidrosis patients receiving treatment not covered by health insurance remained unknown. Therefore, only the cost per patient was evaluated instead of making a nationwide estimate. This should be further considered because the out‐of‐pocket expenses for treatments not covered by health insurance cannot be ignored in the evaluation of the medical cost associated with axillary hyperhidrosis. Moreover, the severity rates in focal hyperhidrosis patients were utilized in our analysis because no data are available on HDSS‐based severity rates in axillary hyperhidrosis patients^18^ We considered it possible to substitute axillary hyperhidrosis because it belongs to focal hyperhidrosis. However, as it will provide important evidence for making nationwide estimation, future investigation is awaited. Although WPAI (%) in each disease level (i.e., HDSS level) was evaluated, the possibility that disease duration also affects the degree of WPAI cannot be denied. Future studies evaluating the relationship between disease duration and WPAI (%) are expected. The Web‐based questionnaire survey was conducted in an emergency state due to the spread of COVID‐19 infection. Therefore, the unusual living environment may have affected the survey. However, the impact on hygiene product costs may have been limited because the recall period of the survey on hygiene costs was set as the past 1 year. The period for estimating the productivity loss was set as 1 month to avoid over‐ and underestimations.

The cost associated with primary axillary hyperhidrosis in Japan was clarified. Active interventions are needed for hyperhidrosis patients because improving the symptoms should reduce the societal loss by increasing productivity and improve the individual’s quality of life.

As estimated in our survey, the direct medical cost per patient with axillary hyperhidrosis in Japan was ¥75 036/year (FY 2018), the annual hygiene product cost was ¥24.5 billion, and the societal loss associated with the productivity loss was ¥312 billion/month. The hygiene product cost and the productivity loss slightly increased at a higher HDSS level. Considering the out‐of‐pocket expenses for treatments not covered by health insurance, the cost associated with axillary hyperhidrosis is even higher.

## CONFLICT OF INTEREST

H.M., T.F., and H.Y. received fees as resource speakers from Kaken Pharmaceutical; H.O. and H.M. are employees of Kaken Pharmaceutical and have a stock in Kaken Pharmaceutical; and S.I. is an employee of CRECON Medical Assessment. CRECON Medical Assessment was paid by Kaken Pharmaceutical to conduct analyses for the study.
